# Novel educational and goal-setting tool to improve knowledge of chronic kidney disease among liver transplant recipients: A pilot study

**DOI:** 10.1371/journal.pone.0219856

**Published:** 2019-07-25

**Authors:** Rachael B. Leek, Jeong M. Park, Claire Koerschner, Jennifer Mawby, Christopher J. Sonnenday, Julie A. Wright Nunes, Pratima Sharma

**Affiliations:** 1 Department of Pharmacy and College of Pharmacy, Michigan Medicine, University of Michigan Ann Arbor, Michigan, United States of America; 2 Department of Surgery, Michigan Medicine, University of Michigan, Ann Arbor, Michigan, United States of America; 3 Division of Nephrology, Michigan Medicine, University of Michigan, Ann Arbor, Michigan, United States of America; 4 Division of Gastroenterology and Hepatology, Michigan Medicine, University of Michigan, Ann Arbor, Michigan, United States of America; Harvard Medical School, UNITED STATES

## Abstract

**Introduction:**

Liver transplant (LT) recipients have limited understanding of post-transplant chronic kidney disease (CKD) despite an excellent pre-existing framework of transplant care. This pilot study examined the efficacy and feasibility of a tailored educational and goal-setting tool in improving CKD knowledge among LT recipients with early-stage CKD.

**Methods:**

In this prospective cohort study, we administered the CKD educational and goal-setting tool to 81 LT recipients between 7/1/2016 and 12/31/2017. We excluded patients with simultaneous liver-kidney transplantation, eGFR<30 ml/min, non-English speaking, on hemodialysis or listed for kidney transplantation. The pre- and post-education knowledge scores were compared using a paired t-test. Linear regression was used to assess the independent predictors of change in knowledge score.

**Results:**

Mean age was 56.3 years, 69.1% were males, 85.2% were Caucasians and mean eGFR was 61.2 ± 20.0 ml/min. The CKD educational and goal-setting tool improved the CKD knowledge scores among LT recipients (pre: 71.8 ± 16.6%, post: 83.3 ± 10.4%; p<0.001). In an adjusted model (r^2^ = 0.75), those with lower pre-education knowledge scores had the most improvement in their post-education knowledge scores (β = -83.2; p<0.001). Two-thirds stated their most important self-management goal and reported motivation to follow this goal. Time spent for the CKD education was approximately 15 minutes.

**Conclusions:**

A simple LT-specific patient educational and goal-setting tool effectively improved CKD knowledge. Implementation of this tailored intervention will improve CKD awareness and may promote goal-setting in the target population.

## Introduction

Chronic kidney disease (CKD) is a major public health problem in the United States affecting 26 million Americans [1, 2]. The total Medicare spending for CKD for aged 65 years and older in 2013 was estimated to be greater than $50 billion [3]. Solid organ transplant recipients are at markedly high risk of developing advanced CKD (stage 4–5 CKD) [4], and have been designated as a “special population” by the Centers for Disease Control and Prevention’s CKD Surveillance Program [2]. Advanced CKD (stage 4 or greater) is associated with high morbidity, increased health care costs and high mortality with the loss of transplant graft [4–6].

Liver transplant (LT) recipients, the largest group of non-renal solid organ transplant recipients, have a five-year cumulative incidence of 18% for advanced CKD [4]. Through our previous work, it is clear that the incidence of stage 5 CKD among LT recipients has substantially risen over the last 14 years resulting in a two-fold increased risk of hospitalization and three-fold higher risk of death compared to those without [5, 6]. The incidence rate of new onset post-transplant CKD stage 5 (end Stage renal disease requiring dialysis or listed for kidney transplant) is 15.0 per 1000-patient year [7]. The CKD stage 5 population represents only the tip of the iceberg. Our previous work demonstrated that LT recipients whose renal function progressed to end stage renal disease (ESRD) had a higher risk of death compared to patients without post-LT ESRD (HR = 3.32, p<0.0001) [5]. The risk of death increased exponentially as estimated glomerular filtration rate (eGFR) decreased below 30 ml/min. A study analyzing the relationship between CKD and mortality following LT found statistically significant hazard ratios for mortality of 2.7 and 5.5 for eGFR 15–29 ml/min and eGFR less than 15 ml/min, respectively [8].

Previous studies in the non-transplant CKD patient population have utilized patient education interventions intended to improve CKD outcomes [9–12]. Several studies conducted in patients with stage 4–5 CKD showed improved patient outcomes after an educational intervention [9–11]. While benefit is observed in late-stage CKD, there are few studies on educational interventions in early-stage CKD to assess the potential of intervening earlier to improve outcomes [12].

Our previous study revealed that although LT recipients with early-stage CKD (stages 1–3) had lower baseline knowledge about CKD compared to the general CKD population, many were interested in improving their knowledge about CKD progression and prevention [13]. We viewed the risk of developing CKD post-LT as an important knowledge gap for many LT recipients. To address this concern, we tailored an existing CKD education and goal-setting tool [14] to LT recipients based upon the results of our previous study. While we recognize that improving a patient’s knowledge about a disease state does not always result in improved patient outcomes, we believe LT-targeted CKD education may lead to improved patient engagement in their health outcomes post-LT and self-management of their risk factors for disease. In this pilot study, we examined the efficacy and feasibility of this LT-targeted education and goal-setting tool in improving CKD knowledge among LT recipients with early-stage CKD.

## Methods

### Study design and population

This was a prospective study in which we performed a CKD knowledge assessment at baseline, administered a CKD educational and goal-setting tool (intervention) and then reassessed the CKD knowledge after the intervention in a cohort of LT recipients. The CKD knowledge was assessed using Kidney Disease Knowledge Survey after LT (KiKS-LT). Since we focused on evaluating the efficacy and feasibility of the CKD educational and goal-setting tool, this pilot study was not designed to test long-term knowledge retention or impact on clinical outcomes. This study was approved by the University of Michigan institutional review board.

Our inclusion criteria were age at LT ≥18 years, received LT between January 1, 2008 and June 30, 2017, and were at least three months post-transplant. Our exclusion criteria were simultaneous liver-kidney transplantation, eGFR<30 ml/min, non-English speaking, on hemodialysis or listed for kidney transplant. A member of the study team approached all eligible patients at the Michigan Medicine Transplant Center during their clinic visits between July 1, 2016 and December 31, 2017, presented the study information and obtained the informed consent. Following the initial survey, patients were provided the intervention and re-administered the KiKS-LT.

**Fig 1:**
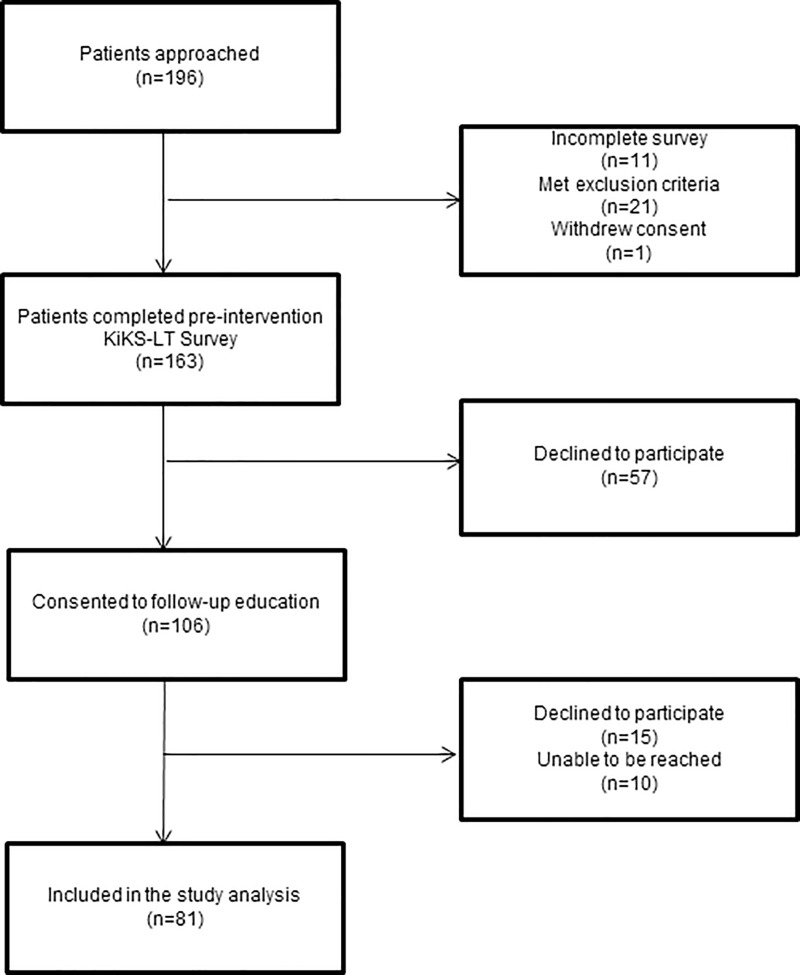
Consort diagram: Cohort determination.

The results of CKD knowledge using KiKS-LT on 163 LT recipients were presented in a separate study [13]. We approached 106 respondents (Fig 1) from our previous study who expressed interest in receiving the personalized educational intervention.

### Patient demographics and baseline characteristics

Of the 106 LT recipients approached to receive education, 15 declined to participate and 10 could not be reached by the research team. Thus, 81 of the 106 patients (76.4%) participated in the educational intervention and completed the post-education KiKS-LT (Fig 1).

Table 1 summarized the baseline patient characteristics. The mean age was 56.3 ± 11.7 years, 85.2% were Caucasian and 69.1% were males. The mean eGFR was 61.2 ± 20.0 ml/min, with almost half of the patients at CKD stage 3. The pre-education survey was performed after a mean of 3.5 ± 3.4 years after LT. The time from the pre-education survey to administration of CKD educational and goal setting tool was a mean of 10.1 ± 3.6 months. The prevalence of hypertension, diabetes mellitus, and a BMI above the normal range were 32.1%, 27.2%, and 72.8%, respectively. The intervention was administered before or after the clinic appointment to 27 (33.3%) participants, and on the telephone to 54 (66.7%) paticipants. The distribution of age, sex, pre-education knowledge scores, eGFR, BMI and systolic BP at baseline were similar between the two groups.

**Table 1:** Baseline patient characteristics (n = 81).

Age, mean ± SD (years)	56.3 ± 11.7
Male sex, n (%)	56 (69.1)
**Race/Ethnicity**	
Caucasian	69 (85.2)
African American	7 (8.6)
Asian	4 (4.9)
Other	1 (1.2)
**Etiology of liver disease**	
Hepatitis C/HCC	31 (38.3)
Autoimmune/PBC/PSC	20 (24.7)
Cryptogenic cirrhosis/NAFLD	12 (14.8)
Alcoholic liver disease	6 (7.4)
Others	12 (14.8)
Hepatocellular carcinoma, n (%)	18 (22.2)
Time from LT to first KiKS-LT, mean ± SD (years)	3.5 ± 3.4
Time from first KiKS-LT to education, mean ± SD (months)	10.1 ± 3.6
Time from first KiKS-LT to end of follow up (months)	16.5 ± 2.5
eGFR, mean ± SD (mL/min)	61.2 ± 20.0
CKD Stage, n (%)	
Stage 1 CKD	6 (7.4)
Stage 2 CKD	35 (43.2)
Stage 3 CKD	40 (49.4)
RRI, mean ± SD	4.5 ± 4.6
Systolic BP, mean ± SD (mmHg)	136.4 ± 19.9
Diastolic BP, mean ± SD (mmHg)	75.1 ± 9.7
Hypertension	26 (32.1)
Diabetes mellitus	22 (27.2)
BMI > 30	33 (40.7)
BMI 25–30	26 (32.1)
BMI Normal	22 (27.2)
**Education**	
High school or less	27 (33.3)
Some college or more	42 (51.9)
Missing	12 (14.8)
Mode of delivery of education, n (%)	
Phone	54 (66.7)
In clinic	27 (33.3)

BMI, body mass index; BP, blood pressure; CKD, chronic kidney disease; eGFR, estimated glomerular filtration rate; HCC, hepatocellular carcinoma; LT, liver transplant; NAFLD, non-alcoholic fatty liver disease; PBC, primary biliary cholangitis; PSC, primary sclerosing cholangitis; SD, standard deviation.

## Survey instrument: Kidney Disease Knowledge Survey after LT (KiKS-LT)

The survey instrument was adapted for LT recipients from the validated Kidney Knowledge Survey (KiKS) [13,14]. The KiKS-LT targeted stage 1–3 CKD and was reflective of the needs of the LT recipient population. The adapted survey consisted of thirty-one questions, the content of which was previously described [13]. In brief, the KiKS-LT examined CKD knowledge in the following domains: 1) general knowledge of kidney disease (11 items); 2) LT-specific CKD risk factors and immunosuppression knowledge (4 items); 3) kidney function (7 items); and 4) symptoms of advanced CKD (9 items). The final question asked whether the patient was interested in learning more about CKD in order to be more active in managing their kidney disease risk factors. Since the goal was to assess the efficacy of our educational intervention and goal-setting tool with respect to LT-specific risk factors and risk modification for prevention of kidney disease, we mainly focused on the 15-survey questions related to knowledge domains 1 and 2 for this study. The KiKS-LT score was calculated using the percentage of questions answered correctly out of 15 total possible questions with one best answer for each question. Scores could range from 0 to 100 percent correct.

## KiKS-LT education and goal-setting tool

**Fig 2:**
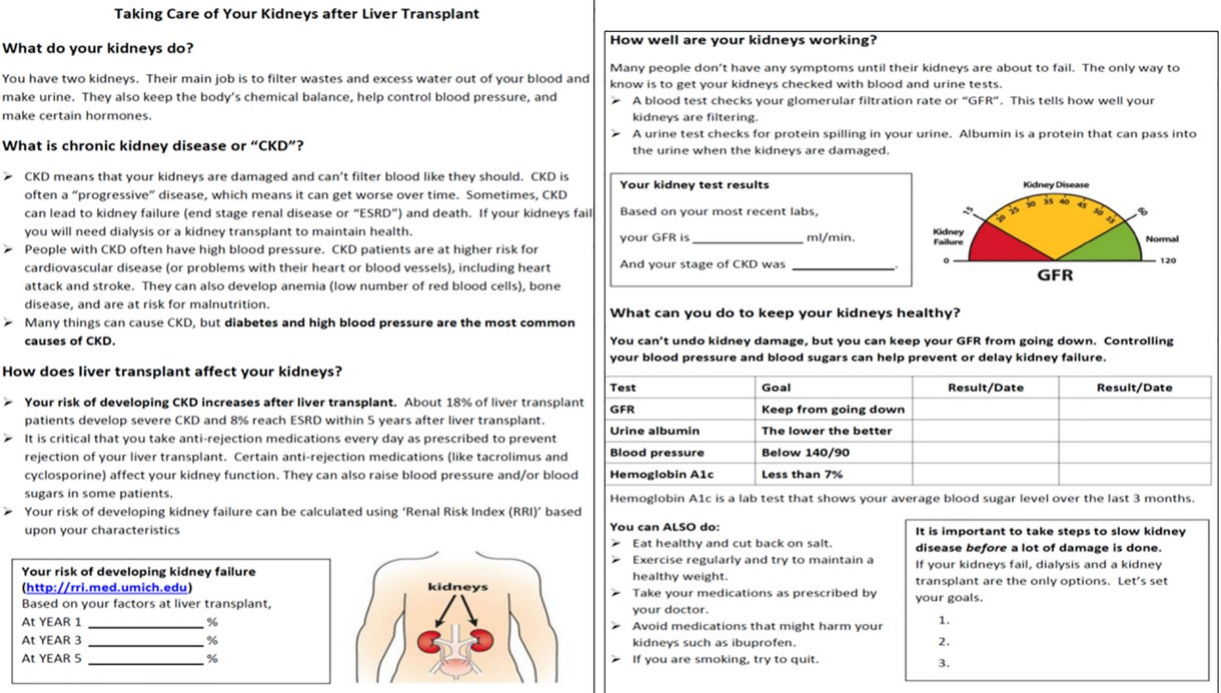
Chronic kidney disease educational and goal-setting tool for liver transplant recipients.

Knowledge assessment and education tools are developed with the intention of identifying knowledge gaps in patients, and creating dialogue between the patient and their health care provider. In turn, this gives providers areas to focus on during educational sessions with their patients. In order to improve the CKD knowledge and promote goal-setting among LT recipients, we tailored an existing CKD education and goal-setting tool^10^ based upon the knowledge gaps identified in the KiKS-LT [13]. The post-LT CKD educational and goal-setting tool (Fig 2) is a single sheet (front and back-sided) handout that covers the basic functions of the kidney, provides an explanation of CKD as well as its causes and comorbidities, and educates patients on their risk of developing CKD and ESRD after LT. Additionally, it provides LT recipients information on monitoring CKD with emphasis on interpreting eGFR as it relates to staging of CKD. In the handout, participants also received a personalized table of their recent eGFR, urine albumin, blood pressure (BP), and hemoglobin A1c (HbA1c) results (if diabetic), and the goals for self management for each result.

Education sessions were provided by either a Pharm D- transplant pharmacist (RL) or a Psychology major bachelor-level study coordinator (CK) to LT recipients post-transplant. To reduce inter-educator variability, we created a standardized, patient-level script to match the above description for the education sessions that were projected to last approximately 15 minutes. The liver transplant physician (PS) was available to answer any additional question(s) posed by the subject(s). The script first introduced the function of the kidneys, the effects of kidney damage and an explanation of eGFR in monitoring kidney disease. The script then explained the LT-specifc risk factors (calcineurin inhibitors) and role of traditional risk factors (hypertension, diabetes and obesity) in CKD progression to ESRD, the importance of medication adherence and goals of management for blood glucose and HbA1c if diabetic, and BP if hypertensive. The final section of the handout included education on maintaining a healthy diet and weight, medication adherence, avoiding nephrotoxic medications, and smoking cessation. Based on the educational points reviewed, participants were provided space to set three goals to slow kidney disease progression. The intention behind the patient goal-setting was to encourage participation in and ownership of managing their risk of CKD progression independently.

In order to help participants understand the post-LT ESRD risk, we calculated their renal risk index (RRI) score [7] (https://rri.med.umich.edu) at the time of education session. RRI is an objective score that accurately predicts the risk of new onset post-transplant ESRD among LT recipients. In addition to reviewing their RRI score (compared to the reference LT recipient with RRI score of 1.0), we also recorded the decile in which their RRI score was. Based upon their decile, we provided the participants with their personalized 1-, 3- and 5-year cumulative incidences of developing post-LT ESRD using RRI calculator [7]. The education was provided by two study team members (RL and CK) using a standardized script. This standardized script was personalized to a patient-level discussion and included their projected risk of developing CKD at 1, 3, and 5 years post-transplant based on the calculated RRI. Senior study team members (PS-transplant hepatologist and JP-transplant pharmacist) were available to answer any question or concern that the study participants had regarding their personalized risk of post-LT ESRD.

Based upon participant’s preference indicated on the pre-education survey after LT, follow-up education sessions were provided either in conjunction with their scheduled clinic visit or by phone. For education in clinic, patients were counseled one-on-one in private consultation rooms and were presented with a copy of the KiKS-LT educational tool at the start of the education. For education by phone, a copy of the KiKS-LT educational tool was sent to the patient by email at the start of the phone conversation and they were instructed to follow along on the device they accessed email with. Immediately following the educational intervention, patients were surveyed again using the KiKS-LT on paper if educated in clinic, or verbally if educated by phone.

## Data collection

The following patient-specific factors were collected from the electronic medical record: demographics, serum creatinine, urine analysis, BP, comorbidities, education level, CKD stage based on eGFR at the time of pre-education survey, education and follow up, RRI, date of LT, time from LT to pre-education KiKS-LT, time from pre-education KiKS-LT to education, whether the patient was followed by a nephrologist, and presence of hypertension or diabetes mellitus at the time of the initial KiKS-LT. The post-education KiKS-LT was completed shortly after the education session, usually the same day, and was collected by the educator. CKD stages were determined based on eGFR calculated by the Modification of Diet in Renal Disease (MDRD-4) equation at the time of the initial KiKS-LT [15]. The de-identified raw data file is listed under supporting information as S1 File.

## Statistical analysis

The continuous and categorical variables were expressed as mean ± standard deviation and percentage, respectively. The primary outcome was change in the KiKS-LT knowledge scores. The pre- and post-education knowledge scores were calculated as the percentage of correct answers on the KiKS-LT by each patient before and after the educational and goal-setting tool was provided. Since the focus of this study was to educate the LT recipients with early-stage CKD on LT-specific CKD risk factors and risk modification for prevention of CKD, the composite pre- and post-education knowledge scores did not include the answers to questions on functions of the kidney and symptoms of advanced CKD. The pre- and post-education knowledge scores were compared using a paired t-test. For each question, answers before and after the educational intervention within the same patient were compared using McNemar’s test.

Next, we examined the factors associated with change between pre- and post-education KiKS-LT knowledge scores using a stepwise linear regression analysis. In particular, covariates of interest included mode of education (clinic vs. phone); age, gender, education, educator type (transplant pharmacist vs. trained bachelor level study coordinator), CKD stage and risk factors of CKD (hypertension, diabetes, BMI and RRI decile). The covariates with p<0.10 were entered in the adjusted multivariate linear regression model.

In a secondary analysis we examined the association between change in the CKD knowledge score and change in eGFR at last follow up using linear regression analysis. This model was adjusted for age, gender, diabetes, systolic BP and change in the KiKS-LT knowledge score. A p-value <0.05 was considered statistically significant for all study findings. All statistical analyses were performed using IBM SPSS version 24 (IBM Corp., Armonk, NY).

## Results

### CKD educational and goal-setting tool

Time spent on CKD education was on average approximately 15 minutes. At the end of the education session, all of the patients were asked to set three self-management goals intended to mitigate the risk of CKD progression. Sixty four percent of the participants set at least one self-management goal (No goal = 36%; 1 goal = 18%; 2 goals = 30%; and 3 goals = 16%) while 36% did not set any goal. While 14% identified better control of hypertension and diabetes as one of their goals, common goals set were a desire to exercise (29%), improve diet/weight loss (29%), limit sodium intake (21%), and increase water intake (11%). The patients who received the education via telephone were more likely to set any self-management goal compared to those who received education in the clinic (91% vs. 4%; p<0.001). Males were more likely to set goals than the females (71% vs. 48%; p = 0.049)

### Knowledge score before and after the intervention

There was a statistically significant increase in the mean KiKS-LT score after the administration of the CKD educational and goal-setting tool (Table 2). When we analyzed the individual LT-specific knowledge questions, we found significant increases in the percentage of participants providing the correct answer to the following questions: 1) LT is a risk factor for CKD (76.5% vs. 98.8%, p<0.001)?, and 2) CKD is a risk factor for heart disease (71.6% vs. 97.5%, p<0.001)?. There were also significant increases in the percentages of participants answering correctly in two diabetes knowledge questions: 1) definition of HbA1c (61.7% vs. 88.9%, p<0.001), and 2) HbA1c goal for good control of diabetes (46.9% vs. 79.0%, p<0.001).

**Table 2:** Comparison of CKD knowledge survey scores between pre- and post-education.

Outcome	Pre-education	Post-education	P-value
KiKS-LT Score (mean ± SD)	71.8 ± 16.6%	83.3 ± 10.4%	<0.001
Selected Individual Questions			
Knowledge of Risk of CKD Post-LT	76.5%	98.8%	<0.001
Knowledge of CKD as a Risk Factor for Heart Disease	71.6%	97.5%	<0.001
Knowledge of HbA1c Definition	61.7%	88.9%	<0.001
Knowledge of Diabetes Goals: HbA1c	46.9%	79%	<0.001

CKD, chronic kidney disease; HbA1c, hemoglobin A1c.

### Factors predicting change in knowledge score

The mean change in the KiKS-LT score from pre- to post-CKD education was 10.7 ± 16.4%. In an adjusted stepwise linear regression model (adjusted R^2^ = 0.75; intercept: 83.2; p<0.001), presence of hypertension (β = 0.038; p = 0.049), intervention given in clinic (β = 0.11; p = 0.005), goal-setting (β = 0.076; p = 0.03), and pre-education KiKS-LT score (β = -83.2; p<0.001) were independently associated with the significant improvement in the CKD knowledge score after the intervention. Each percent decrease in pre-education KiKS-LT score increased the change in post-education survey score by 83% on an average.

### Factors associated with change in eGFR

The mean change in eGFR from the date of pre-survey to last follow up was 2.9 ± 26.2 ml/min. In an adjusted model, age (p = 0.005) and systolic BP (p = 0.012) were the independent predictors of change in eGFR, but the change in KiKS-LT score was not (Table 3).

**Table 3:** Independent predictors of change in eGFR from the first durvey to end of follow up.

Covariate	Beta (95% CI)	p-value
Intercept	13.2 (-31.3, 57.7)	0.56
Age (per year)	0.72 (0.23–1.214)	0.005
Gender	1.76 (-10.6, 14.15)	0.8
Systolic BP (per 10 mmHg increase)	-3.69 (-6.53, -0.85)	0.012
Change in KiKS-LT score	-5.18 (-40.7, 30.63)	0.8
Diabetes mellitus	-4.0 (-17.3, 9.3)	0.55

BP, blood pressure; eGFR, estimated glomerular filtration rate.

## Discussion

This is the first study to our knowledge that tested the efficacy of a CKD educational and goal-setting tool among LT recipients with early-stage CKD. There was a significant improvement in the mean KiKS-LT knowledge score after the educational intervention, indicating that the educational tool was effective in improving CKD knowledge in the post-LT population. Most improvement in the knowledge after the intervention was seen in those with lower KiKS-LT score with hypertension, who received intervention in the clinic and did the goal-setting. Additionally, the magnitude of knowledge improvement had no significant association with time from LT, CKD stage or type of educator (transplant pharmacist or bachelor level study coordinator). This suggests that the CKD education used in this study can be effectively completed using the standardized script regardless of time from LT.

A validated non-transplant patient knowledge survey (KiKS) revealed that even when a patient has established care with a nephrologist, their knowledge about their CKD diagnosis and about the the kidney is limited [14]. Additional research has shown that when nephrology fellows provided individualized, CKD-specific education to patients using a similar educational tool, there was no significant improvement in overall summary scores of knowledge comparing patients with CKD who received the intervention to a historical cohort [16]. However, when examining individual domains about CKD, improvement was noted: patients who received the intervention better understood their CKD diagnosis, identified their own eGFR, and knew that there were stages of CKD [16]. Although a direct comparison to our study is not possible, our research did find a significant improvement in the overall CKD knowledge in a cohort of LT recipients after they received our educational tool. This supports that in the LT patient population, an early educational intervention including goal-setting and encouraging patient ownership of preventing CKD progression is effective at improving patient knowledge, even as assessed as an overall summary measure, about CKD. In addition, we found that our educational tool increased the participants’ awareness and knowledge about several topics of critical importance in CKD—namely patients were more aware of the increased risk of CKD after LT, knew that heart disease could be associated with having CKD, and better understood goals of CKD risk management, such as their HbA1c goal.

Lack of knowledge is a barrier to patient engagement and self-management of chronic disease risk factors. Well-designed interactive educational interventions not only can improve patient’s knowledge but also foster patient engagement in self-management toward CKD risk factor management [10,12,16,17]. It is encouraging that during educational tool delivery, the majority of our study partipants set goals to work toward lifestyle modifications that may decrease their risk for CKD progression, a first and important step to actualizing behavior change. Lifestyle modification in terms of healthy eating, limiting sodium intake, increasing hydration and exercise were the most common goals.

Our study was not designed to examine a long-term clinical impact lasting beyond the improvement in the CKD knowledge score. Given the small sample size and short duration of follow up of our study, it was not surprising that improvement in knowledge score and goal-setting did not affect the change in eGFR in our study. It is encouraging that a previous study of an educational intervention in CKD that found a significantly extended time to dialysis initiation (median 17.0 months vs. 14.2 months, p<0.001) [11]. Another measured a significantly better eGFR in the intervention group compared to standard of care (29.1 ml/min vs. 15.7 ml/min, p<0.05) [10]. Future studies need to evaluate whether improved understanding of CKD can enable LT recipients to be more involved in their care with respect to prevention and risk factor modification that may mitigate CKD progression.

The limitations include single center study design, high baseline education level of participants (51.9% of participants had at least some college education), and potential for selection and recall bias—as the post-education KiKS-LT was performed shortly after the education session. Beyond level of education completed, we were unable to assess other findings such as health literacy, numeracy (or quantitative literacy, refers to the ability to access, understand, and apply numerical data to health-related decisions), or cognitive function of the participants, which each may have influenced their baseline knowledge of CKD as well as their ability to complete the KiKS-LT. Using two separate modes of education may have biased the participant toward setting more goals because they were verbally asked to provide goals rather than instructed to write them down. As this was a feasibility assessment of a one-time intervention, we do not know whether an educational tool would provide persistent CKD knowledge or whether the information impacts patient outcomes. Studies assessing for knowledge retention after a CKD educational session in LT recipients are warranted. However, our study was conducted at a center with a liver transplant population similar to the median across the U.S., adding support to the potential for applicability of our study results to similar transplant centers.

In conclusion, this study demonstrated that the provision of a patient-specific CKD educational and goal-setting tool to LT recipients with early-stage CKD was effective at improving patient knowledge about CKD. This CKD educational and goal-setting tool may benefit larger populations beyond LT recipients with preserved renal function. Future studies are needed to apply this successfully not only to LT recipients, but potentially to renal and other non-renal organ transplant recipients as well.

## Supporting information

S1 File Deidentified data file: Plos_one_data. (XLSX)

## Abbreviations

BMI: Body Mass Index
BP: Blood Pressure
CKD: Chronic Kidney Disease
CI: Confidence Interval
eGFR: Estimated Glomerular Filtration Rate
ESRD: End Stage Renal Disease
HbA1c: Hemoglobin A1c
HR: Hazard Ratio
KiKS: Kidney Disease Knowledge Survey
LT: Liver Transplantation
